# Measuring medical students’ professional competencies in a problem-based curriculum: a reliability study

**DOI:** 10.1186/s12909-019-1594-y

**Published:** 2019-05-21

**Authors:** Salah Eldin Kassab, Xiangyun Du, Egon Toft, Farhan Cyprian, Ayad Al-Moslih, Henk Schmidt, Hossam Hamdy, Marwan Abu-Hijleh

**Affiliations:** 1 College of Medicine, Qatar University, PO Box 2713, Doha, Qatar; 2 College of Education, Qatar University, Doha, Qatar; 3 Faculty of Social Sciences, Erasmus University, Rotterdam, Netherlands; 4 Gulf Medical University, Ajman, United Arab Emirates; 5 UNESCO Center for PBL, Aalborg University, Aalborg, Denmark

**Keywords:** G-theory, Problem-based learning, Professional competencies, Reliability, Student assessment

## Abstract

**Background:**

Identification and assessment of professional competencies for medical students is challenging. We have recently developed an instrument for assessing the essential professional competencies for medical students in Problem-Based Learning (PBL) programs by PBL tutors. This study aims to evaluate the reliability and validity of professional competency scores of medical students using this instrument in PBL tutorials.

**Methods:**

Each group of seven to eight students in PBL tutorials (Year 2, *n* = 46) were assessed independently by two faculty members. Each tutor assessed students in his/her group every five weeks on four occasions. The instrument consists of ten items, which measure three main competency domains: interpersonal, cognitive and professional behavior. Each item is scored using a five-point Likert scale (1 = poor, 5 = exceptional). Reliability of professional competencies scores was calculated using G-theory with raters nested in occasions. Furthermore, criterion-related validity was measured by testing the correlations with students’ scores in written examination.

**Results:**

The overall generalizability coefficient (G) of the professional competency scores was 0.80. Students’ professional competencies scores (universe scores) accounted for 27% of the total variance across all score comparisons. The variance due to occasions accounted for 10%, while the student-occasion interaction was zero. The variance due to raters to occasions represented 8% of the total variance, and the remaining 55% of the variance was due to unexplained sources of error. The highest reliability measured was the interpersonal domain (G = 0.84) and the lowest reliability was the professional behavior domain (G = 0.76). Results from the decision (D) study suggested that an adequate dependability (G = 0.71) can be achieved by using one rater for five occasions. Furthermore, there was a positive correlation between the written examination scores and cognitive competencies scores (r = 0.46, *P* < 0.01), but not with the other two competency domains (interpersonal and professionalism).

**Conclusions:**

This study demonstrates that professional competency assessment scores of medical students in PBL tutorials have an acceptable reliability. Further studies for validating the instrument are required before using it for summative evaluation of students by PBL tutors.

## Background

The emergence of competency-based education had led to identification of the core professional competencies that medical students should demonstrate at the point of graduation [1, 2]. However, many domains of competence received limited attention in the competency models, especially those related to professional behavior including attitudes, humanity, personal values, responsibility, reflection, and responding to events as they get disclosed [3, 4]. Specifically, measuring the general professional competencies such as the ability to work in a team, professional behavior, or cognitive and metacognitive skills, has been a challenge [5, 6].

The challenge in measuring the so-called “difficult-to-measure” professional competencies can be attributed to multiple factors. Firstly, the prevailing assessment methods tend to isolate the competencies to be measured into smaller units instead of examining the subject as a whole person or professional, with the assumption that grasping the parts will spontaneously lead to incorporated competent performance [6]. Secondly, the majority of methods existing in the literature are based on self-reported questionnaires or assessment of peers. Unfortunately, self-assessment scores of professional competencies do not correlate with external measures of performance [7, 8]. Furthermore, the psychometric properties of the currently existing tools for peer assessment do not provide conclusive evidence [9, 10]. Thirdly, many of the existing methods take place in the context of postgraduate clinical training and tend to focus on trainee or observer attitudes about the assessment tool rather than measuring the actual performance [11]. These challenges call the need for devising longitudinal, course-independent measures to evaluate multiple competencies using one assessment method [6].

We have recently developed an instrument composed of a list of the most important profession-related competencies that medical students should develop during the course of their study in order to prepare for their future professional practice [12]. The instrument was developed through a comprehensive literature review, followed by two rounds of input from a group of medical education experts using the Delphi model. These competencies were grouped under three main domains namely cognitive, interpersonal and professionalism domains. However, the evidence for the reliability and validity inferences from this instrument was not demonstrated. In PBL tutorial groups, students and tutor work closely together. This learning atmosphere is conducive to foster and monitor appropriate professional behaviors of students [13]. The longitudinal observation of students by tutors in PBL tutorials provides a valuable opportunity to observe and assess the development of students at the levels of cognitive, personal and interpersonal domains. Therefore, we aimed to explore possibilities of measuring what are often regarded as difficult-to-measure competencies of medical students by PBL tutors through longitudinal observation in PBL tutorials.

This study, therefore, is designed to answer the following formulated research questions:

1. What is the reliability of the medical students’ essential professional competencies scores using the study instrument?
2. To what extent can we generalize the medical students’ scores in essential professional competencies across PBL raters and occasions?
3. What is the predictive validity of the students’ scores in essential professional competencies in relation to their scores in other related domains of competence?

## Methods

### Design and study setting

This is a generalizability theory analysis, which evaluates the reliability of the assessment scores of essential professional competencies of medical students. The study was conducted at the College of Medicine, Qatar University (CMED-QU), which commenced its medical program recently (in 2015). The medical program spans across six years divided into three phases: Phase I (one year), Phase II (two and a half years), and Phase III (two and a half years). The first phase is primarily lecture-based, involving teaching of fundamental science courses. The second phase is composed of twelve integrated body-system units using PBL as the main educational strategy. The third phase consists of clinical rotations in hospitals and primary health care centers. During each week of phase II, students study a problem in small groups and each problem is designed to cover learning objectives from basic medical sciences, clinical sciences, and population medicine. PBL tutorials are conducted in two sessions per week (2.5 h each) with faculty members acting as facilitators. At the end of each PBL unit, students undertake a summative integrated written (MCQs) examination. Furthermore, at the end of each semester, students undertake a summative integrated Objective Structured Practical Examination (OSPE) and Objective Structured Clinical Examination (OSCE) in the different PBL units that have been studied during the semester.

### Study participants

This study was conducted with year 2 medical students (*n* = 46) during their study in the PBL units for the academic year 2016–2017. Students were divided into six PBL tutorial groups. Each group comprised of a mix of seven to eight male and female students. The group stayed together throughout the academic year. Each tutorial group was assigned two PBL tutors. At the end of each unit, the two PBL tutors independently assessed the essential professional competencies of each individual student in a group, based on longitudinal observation of the students during the PBL tutorials for around 5 weeks. The PBL tutors filled the evaluation form on four occasions: at the end of Unit II (Body Defense), Unit III (Cardiovascular system), Unit IV (Hematopoietic system), and Unit V (Respiratory system). Prior to the study, all PBL tutors attended a workshop that explained the purpose of the study and the process of filling the questionnaire in order to standardize the process.

### Measures

The study instrument consists of ten items representing evaluation of essential professional competencies for medical students in the medical program. The instrument was developed from a recent study following a modified Delphi approach [12]. First, a list of 46 crucial profession-related competencies were identified from extensive literature review and a workshop with medical education experts. The list was then shortened to 26 items through the first round of the modified Delphi survey (feedback questionnaire) by an international panel consisting of 12 experts. The second round of the modified Delphi survey was conducted by a group of PBL tutoring faculty (*n* = 18) at the College of Medicine- Qatar University and yielded ten items clustered under three domains. The first essential professional competency domain of the instrument used represents cognitive competencies such as problem solving, critical thinking and reflectivity. The second domain is interpersonal competencies such as communication and collaboration. The third domain is professional behavior, which included integrity, sense of responsibility, respect, empathy, and time management. Tutors assessed students on each item, based on a 5-point Likert scale (1 = Poor, 2 = Below expectations, 3 = Meets expectations, 4 = Exceeds expectations, 5 = Exceptional). Possible overall student mean scores on each occasion by each rater was therefore between 1 and 5 points.

### Data analysis

#### G-theory analysis

In order to test the reliability of the study instrument, we used the generalizability theory (G-theory) analysis. With data collected in this study, an observed student measurement can be decomposed into a component for the student based on their *universe score* (expected value of the student’s observed scores over all possible observations in the study) and one or more *facets* (which are error components). The object of measurement, here students, is not a source of error and, therefore, is not a facet. This method simultaneously takes into account the various sources of error, which affect the measurement rather than assuming one source of measurement error.

For G-theory analysis, we use ANOVA methods to decompose variance and then compute the variance components by method of moments. The variance components are then used to define reliability coefficients (in the form of an intraclass correlation) to define agreement between a student observed score (under the various conditions) and universe score. The references to the equations and methods have been detailed in previous studies [14–16].

### Study facets

This method partitions the variance in scores to that due to study subjects (*objects of measurement*) and to the various *facets* of measurement and of course the remaining is unexplained variance. Accordingly, using the G-theory analysis in the current study allows the estimation of the variances attributed to differences between students, variance due to differences between the ratings of PBL tutors (two raters per group), and variance due to differences across occasions (four occasions). In addition, this method allows the estimation of the variance due to interactions between students’ scores, and measurement occasions but not PBL raters as they were nested within occasions.

Generalizability studies generally make use of random facets, these being a set of conditions randomly sampled from the universe of conditions for that facet, considered exchangeable with any other sample. On the other hand, fixed facets are rarely used and if they are included and their conditions are not exchangeable then a separate G study would be required for each condition of that facet. The facets in this study were all random since we are interested in generalizing the study findings beyond the context used in the current study and thus raters and occasions are considered exchangeable.

We undertook a nested G-study design as opposed to a crossed design. Fully crossed designs have all objects of measurement (students) rated by all raters on all occasions. In our design, we have nested raters within occasions because different PBL raters were used on each of the four occasions of the study. We have also analyzed the standard error of measurement (SEM), which is used to calculate a range of values around the students score with a specified probability (95%) for this range to include the students “true” mean score. Thus the SEM is especially useful in determining the degree of precision with which student measurements are made using the scale in a particular way (i.e. two raters and four occasions) [14].

Typically, the G-theory analysis includes both the generalizability study (G-study) and the decision study (D-study). The G-study test the different aspects of measurement variance attributed to the facets of the study (raters and occasions). The D-study tests how the generalizability coefficient can change under different facet conditions, and therefore, how best to optimize the measurement [14]. Therefore, the D-study was conducted to predict the optimum number of raters and occasions that are required to achieve an acceptable generalizability coefficient. The minimum acceptable generalizability coefficient (G-coefficient) applied in this study was 0.7 as previously reported [17].

### Predictive validity and temporal stability

The ability of the students’ scores in professional competencies to predict their scores in the written examinations was measured using the *Pearson’s Product-moment* correlation coefficient, and the strength of the correlation was interpreted based on standard recommendations [18]. Furthermore, we used a one-way repeated measures ANOVA to examine the differences between the students’ scores in professional competencies across the four occasions of the study. *Post-hoc* pairwise comparisons between students’ scores across the four occasions was conducted using a paired *t-test* utilizing the Bonferroni correction for multiple testing.

The data were analyzed using IBM SPSS Statistics for Windows Version 23.0 (IBM Corp., Armonk, NY, USA). The G-theory analyses were conducted by using the G1.sps program as previously described [19]. The variance components were computed using the *ANOVA* matrix algorithm [20]. Data were presented as the mean ± SD of each parameter and a *p*-value of < 0.05 was considered to indicate differences of statistical significance.

## Results

### Overall reliability of the study instrument

The G-study finds, a relative reliability (consistency) of the scores of professional competences across the two study facets (consisting of 2 raters and 4 occasions) of 0.80 (Table 1). Thus, the observed scores and universe scores were consistent while absolute reliability was less so. In the current study, the percentage of variance attributable to the object of measurement, students, is 27% of total variance and while this is low, the reliability (consistency) of the scores was improved by having more than one rater and occasion.

**Table 1:** Generalizability theory study (G study) results for the scores of medical students (*n* = 46) in professional competencies using two PBL tutors as raters and four measurement occasions in PBL tutorials

*Facets*	*df*	*SS*	*MS*	*Variance component*	*Percent variance*
*Students (s)*	45.00	51.10	1.14	0.12	27.0%
*Occasion (t)*	3.00	16. 88	5.63	0.04	10.0%
*Rater (r): t*	4.00	7.23	1.81	0.03	8.0%
*s × t*	135.00	26.83	0.20	0.00	0.0%
*s × r:t*	180.00	42.48	0.24	0.24	55.0%
*G coefficient: 0.80*					
*Absolute SEM: 0.21*					

*SS* Sum of squares, *MS* Mean of squares, *df* Degree of freedom, *SEM* Standard error of measurement. The proportion of observed variance explained by each facet is calculated by dividing the individual variance component by the total observed variance

The percentage variance component for occasions represented 10% of total variance suggesting that the tests on different occasions differ somewhat in average difficulty, and to a similar extent (8% explained variance) the raters differed in average stringency. On the other hand, the interaction between students and occasions of the study was zero, indicating that the relative ordering of student scores do not differ when tested on different occasions. Finally, the three-way interaction among students, raters and occasions (0.24) represented 55% of total variance. This component represents both the variance attributable to the three-way interaction and residual variance attributable to unmeasured facets and this was the largest variance component.

The SEM for the study model using two raters and 4 occasions was 0.21. Therefore, this results in a confidence interval of ±0.41 and the precision of an observed score is about half a point.

Regarding the three professional competency domains, the reliability of the scores across the study facets (2 raters and 4 occasions) were 0.84, 0.76 and 0.76 for interpersonal competencies, cognitive competencies and professional behavior, respectively (Table 2). In addition, the highest percentage of variance attributable to the object of measurement (students) was for interpersonal competencies (35.0%), followed by cognitive competencies (26.5%) and then professionalism competencies (22.3%).

**Table 2:** Generalizability theory study (G study) results for the scores of medical students (*n* = 46) in the interpersonal competencies, cognitive competencies and professional behavior using two PBL tutors as raters and four measurement occasions in PBL tutorials

Competencies	% Variance explained					G- Coefficient	SEM
	Subjects (s)	Occasions (t)	Rater(r): t	s * t	s * r:t		
Interpersonal competencies	35.0%	8.5%	3.5%	0.0%	53.0%	0.84	0.25
Cognitive competencies	26.5%	1.5%	0.0%	5.0%	67.0%	0.76	0.23
Professional behavior	22.3%	15.3%	11.8%	5.6%	45.0%	0.76	0.21

*SEM* Standard error of measurement

### Generalizability across the PBL raters and occasions

**Fig. 1:**
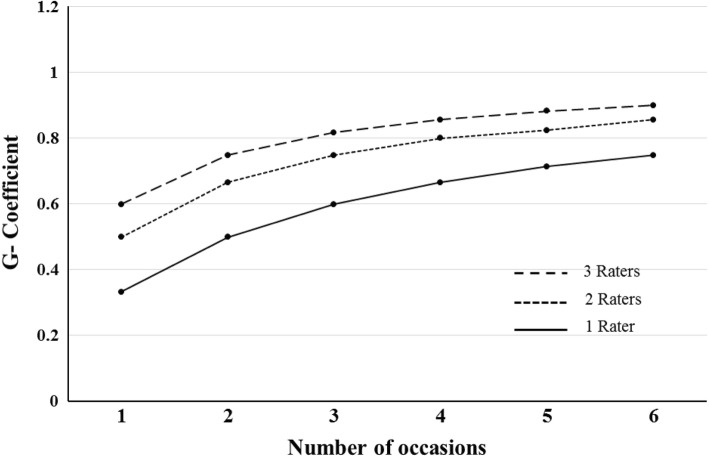
Decision study (*D study*) results for the medical students (*n* = 46) scores in professional competencies. Students were scored using three PBL tutors as raters and six measurement occasions in PBL tutorials. The coefficients are the projected G-coefficient for different combinations of raters and occasions

Figure 1 illustrates the decision (D) study, which predicted reliability of the instrument by using combinations of tutors and occasions. When utilizing a single PBL tutor rater, the number of occasions required for achieving a dependable estimate (G = 0.71) was four occasions. However, using two different PBL raters across four occasions lead to increasing levels of dependability (G = 0.80), and to G = 0.83 when two raters are used across five occasions. As illustrated in Fig. 1, adding a second, third, or fourth occasion resulted in improvements to overall dependability; however, the rate of improvement appeared to diminish beyond four occasions. This flattening in the rate of improvement suggests a point of diminishing returns beyond which additional data collection may not be valuable.

The variance component of 10% for occasions, using two raters, indicates that additional increases in the number of occasions are important for improving the reliability of the instrument. However, the percentage variance component for occasions represented 8.5, 1.5 and 15.3% of total variance for interpersonal competencies, cognitive competencies and professional behavior, respectively suggesting little difference across occasion of measurements on the cognitive domain.

### Predictive validity

To examine the predictive validity of the instrument, we tested the relationship between the scores of students in professional competencies using the study instrument with their scores in written (MCQs) examinations. There were moderate positive correlations between the written examination scores and cognitive competencies scores (r = 0.46, *P* < 0.01), but not with the other two competency domains (interpersonal and professionalism).

### Longitudinal changes in professional competencies

There is a significant main effect of the number of occasions on the scores of cognitive competencies (F (3, 135) = 7.86, *P* < 0.01, ηp2 = 0.15) (Table 3). Students’ scores of cognitive competencies were significantly higher in the 3rd and 4th occasions compared with the 1st occasion (Bonferroni post hoc test). In addition, there was a significant main effect of the number of occasions on the scores of interpersonal competencies (F (3, 135) = 10.61, *P* < 0.01, ηp2 = 0.19). Students’ scores of interpersonal competencies in the first occasion were significantly higher in the 3rd occasion, but not in the 4th occasion, compared with the 1st occasion. Furthermore, there was a significant main effect of the number of occasions on the scores of professionalism competencies (F (3, 135) = 35.55, P < 0.01, ηp2 = 0.44). Students’ scores of professionalism competencies in the 2nd, 3rd and 4th occasions were significantly higher compared with the 1st occasion (*p* = 0.02, P < 0.01 & P < 0.01, respectively). Results therefore suggest that student scores do change temporally but the mean differences do not suggest that this is a practically important difference.

**Table 3:** Differences between mean scores of medical students’ (n = 46) professional competencies in four occasions in PBL tutorials. Data were analyzed by one-way repeated measures ANOVA and using Bonferroni test for post hoc comparisons. Data are presented as mean ± standard deviation of the mean scores

Competencies	Number of occasions				Effect size (ηp2)
	Occasion 1	Occasion 2	Occasion 3	Occasion 4	
*Interpersonal competencies*	3.87 ± 0.84	4.00 ± 0.47	4.35 ± 0.36^**^	4.11 ± 0.54	0.19
*Cognitive competencies*	3.74 ± 0.52	3.89 ± 0.52	4.02 ± 0.60^*^	4.11 ± 0.54^**^	0.15
*Professional behavior*	3.77 ± 0.49	3.97 ± 0.47^*^	4.35 ± 0.36^**^	4.34 ± 0.41^**^	0.44

* = *p* < 0.05 and ** = *p* = < 0.001

## Discussion

We demonstrated that measuring students’ professional competencies can achieve a good level of reliability (G = 0.80) when using two different PBL tutors on four occasions, and an acceptable level of reliability (G = 0.75) when using one PBL tutor on five occasions. In addition, the interpersonal competency domain was the most reliably measured, while professionalism was the least reliable. These findings demonstrate that tutors’ ratings of essential professional competencies of medical students in PBL tutorials demonstrate an evidence of reliability of the assessment instrument used in the study.

The 27% percent variance for the subject of measurement indicates that, averaging over raters and occasions, medical students differed systematically in their professional competency scores. Another supporting evidence for the utility of the obtained ratings is the size of the confidence interval (0.41), given the ratings are being conducted using a 5-point scale. These findings suggest an acceptable degree of variability in tutors’ ratings of student competencies due to unsystematic sources of error. The score variance related to interpersonal competencies domain (communication and collaboration) was the highest (35%) and was to a relatively lesser extent in the cognitive competencies domain (27%). However, the tutor ability to discriminate between students’ levels in professionalism competency domains (22%) was less striking. Ideally, the variance attributable to differences among individuals (i.e., the object of measurement) should contribute the largest proportion than other facets of the study. However, we consider that the variance (22–35%) attributed to the object of measurement in the current study is not surprising considering the difficulty of measuring “difficult to measure” competencies such as responsibility, empathy and integrity*.* Furthermore, an acceptable G-coefficient has been achieved and the variance improved more by increasing the number of occasions. Expanding the study instrument by including items which represent each of the study constructs could lead to a higher percent of variance for the student scores and will be explored in future studies.

This is the first study that used G-theory analysis in measuring students’ professional competencies scores as assessed longitudinally by PBL tutors, which does not allow a comparison with other similar studies. In PBL programs, most of the evidence in the literature demonstrates that evaluation of the professionalism and interpersonal behavior of medical students is mainly conducted through peer assessment in PBL tutorials [21–24]. Only few studies have used G-theory for measuring the reliability of professional competencies scores of medical students by using peer assessment in PBL tutorials [22–24]. However, the reliability and validity of scores for assessing professional behavior of peers in PBL tutorials have been controversial [10]. While the results of the G-theory analysis in our study demonstrated reasonable reliability for the study instrument, further studies in other PBL programs are required to determine the reliability and validity of this instrument beyond the current setting.

The current study demonstrates that the facet of occasion contributes 10% to the model variance, suggesting that this is a good temporal means to improve reliability of measurements of professional competence using PBL tutor ratings. Looking at the percent of variance attributed by different competency domains, rating of interpersonal competencies was the most temporally stable, and professionalism was the least stable across occasions. The 15% variance attributed to occasions in the professionalism domain indicates that the tutor rating of students’ professional behavior in PBL tutorials may require a larger number of occasions to achieve a dependable estimate of professional behavior. On the other hand, the interaction between students and occasions contributed to 0.0% of variance, suggests that the rank order of students did not change dramatically across occasions and there were small changes in rating behavior across occasions.

The variance component for raters nested within occasions represents 8.0% of the total variance. The presence of a nested model in the G-study limits the meaningful interpretation of some facets (and their interactions). In the current study, because we used this nested model, it was impossible to estimate the percentage of variance attributable to differences between raters from the variance attributable to the interaction between raters and occasions independently. Finally, the largest source of measurement error reflected by the three-way interactions (student, rater and occasion), suggests that a large proportion of the variability was caused by facets not included in the study or by random error.

The current findings of the D-study demonstrated that increasing the number of raters from one to two over four occasions resulted in increasing levels of reliability ranging from G = 0.71 to G = 0.80, respectively. However, the requirement for a minimum of two different PBL tutors paired together across several occasions to achieve higher reliability may pose practical constraints in terms of human resource utilization in PBL medical programs. Therefore, increasing the number of occasions using a single PBL tutor appears to be practically more feasible.

In the current study, only the cognitive competencies scores significantly correlated with students’ scores in written examination. Furthermore, the significant increase in the scores of the three competency domains across the four occasions indicates the potential sensitivity of this instrument in revealing the changes in students’ competencies as they progress in the program. We have also recently reported the steps of development of the study instrument using modified Delphi technique [12]. These findings support an evidence of content validity for the study instrument in measuring professional competencies of medical students*.* However, validity is viewed as a structured argument in which evidence is assembled to support or refute proposed interpretations of results [25]. Therefore, there is a need for further studies to examine other lines of validity-evidence such as confirmatory factor analysis or Rash model. Other studies are required for examining whether interpersonal and professional competencies can correlate with students’ performance in other forms of assessments such as academic portfolio and clinical skills exams (OSCE, Mini-CEX, direct observation, etc.).

This study has some limitations that deserve to be reported. The study is limited on its application to year 2 medical students, and large-scale studies are needed to test the replication of our findings in different years of study in other educational settings such as the clinical environment, and in other cultures. Furthermore, there are potential differences in subjective interpretation of the evaluation items by the PBL tutors, especially the items related to professional behavior. Finally, while the validity evidence was tested by correlations with students’ scores in written examination, future studies should test the correlations with students’ performance in other assessments such as PBL tutorial evaluation, OSCE, and student portfolio.

## Conclusions

This study provides a reliability evidence of a tool to be used by PBL tutors for longitudinal assessment of students’ essential professional competencies in PBL programs. A good G-coefficient value (≥ 0.8) could be achieved by having two PBL tutors scoring students over four different occasions and an acceptable value (≥ 0.7) could be achieved by having one PBL tutor who scores students over five different occasions. Tutors can discriminate between the students’ levels of essential professional competencies particularly in the interpersonal domain and less in cognitive and professionalism domains. Further studies will be required to replicate these findings in other contexts before using it for summative assessment of students by PBL tutors.

## Abbreviations

ANOVA: Analysis of variance
D study: Decision study
G study: Generalizability study
G: Generalizability coefficient
MCQs: Multiple choice questions
Mini-CEX: Mini-clinical evaluation exercise
OSCE: Objective structured clinical examination
OSPE: Objective structured practical examination
PBL: Problem-Based Learning
